# The Infestation of Olive Fruits by *Bactrocera oleae* (Rossi) Modifies the Expression of Key Genes in the Biosynthesis of Volatile and Phenolic Compounds and Alters the Composition of Virgin Olive Oil

**DOI:** 10.3390/molecules27051650

**Published:** 2022-03-02

**Authors:** Andrés Notario, Rosario Sánchez, Pilar Luaces, Carlos Sanz, Ana G. Pérez

**Affiliations:** Department of Biochemistry and Molecular Biology of Plant Products, Instituto de la Grasa, Consejo Superior de Investigaciones Científicas, Campus UPO, Ctra. Utrera km 1, Bldg. 46, 41013 Seville, Spain; agprduran@gmail.com (A.N.); rsanchez@ig.csic.es (R.S.); pluaces@ig.csic.es (P.L.); carlos.sanz@ig.csic.es (C.S.)

**Keywords:** olive, virgin olive oil, volatile compounds, phenolic compounds, polyphenol oxidase, lipoxygenase, β-glucosidase, *Bactrocera oleae*

## Abstract

*Bactrocera oleae*, the olive fruit fly, is one of the most important pests affecting the olive fruit, causing serious quantitative and qualitative damage to olive oil production. In this study, the changes induced by *B. oleae* infestation in the biosynthesis of volatile and phenolic compounds in olive (cvs. Picual, Manzanilla, and Hojiblanca) have been analyzed. Despite cultivar differences, the oils obtained from infested fruits showed a significant increase in the content of certain volatile compounds such as (*E)*-hex-2-enal, ethanol, ethyl acetate, and β-ocimene and a drastic decrease of the phenolic contents. The impact of those changes on the inferred quality of the oils has been studied. In parallel, the changes induced by the attack of the olive fly on the expression of some key genes in the biosynthesis of volatile and phenolic compounds, such as lipoxygenase, β-glucosidase, and polyphenol oxidase, have been analyzed. The strong induction of a new olive polyphenol oxidase gene (*OePPO2)* explains the reduction of phenolic content in the oils obtained from infested fruits and suggest the existence of a PPO-mediated oxidative defense system in olives.

## 1. Introduction

Virgin olive oil (VOO) has an exceptional aroma and flavor that distinguishes it from other vegetable oils. The assessment of these two properties by means of a specific sensory analysis is an essential requirement for the commercial classification of virgin olive oils [1]. For this purpose, expert tasters evaluate the green and fruity aroma notes, typical of freshly extracted VOO, and the intensity of the bitter and pungent taste notes of the oils. Volatile and phenolic compounds are responsible for these aroma and taste notes [2,3]. Both types of compounds are produced during the industrial oil extraction process as a consequence of the destruction of the cellular integrity of the olive fruit that triggers a number of biochemical reactions. The volatile and phenolic content of VOO is genetically determined but it is also affected by a number of factors, such as the ripening index of the olive fruit, the geographical origin and the climatic conditions, the technological parameters used during the extraction process, and also the storage time and conditions [4,5,6,7]. It is important to point out that phenolic compounds not only determine the flavor of the VOO, since due to their antioxidant activity they also contribute to its well-known health benefits and improve the oxidative stability of the oils by reducing lipid peroxidation [8,9]. In addition to being decisive for the organoleptic properties of VOO, both volatile and phenolic compounds are involved in the plant’s natural defense mechanisms. This is why their biosynthesis pathways are especially affected by different abiotic (salinity, drought, extreme temperatures) and biotic factors (insects, bacteria, fungi, or viruses) [10,11].

Three decades have passed since we reported [2] that the main volatile compounds responsible for the unique aroma of VOO are synthesized when enzymes from the lipoxygenase (LOX) pathway and their substrates, linoleic (LA) and linolenic acids (LNA), come into contact during the olive fruit milling in the first stage of the oil extraction process. LOX catalyzes the production of 13-hydroperoxide derivatives in the first step of this pathway, which are later cleaved heterolytically by the hydroperoxide lyase (HPL) enzyme, forming six straight-chain carbons (C6) aldehydes [12] that are reduced by alcohol dehydrogenase enzymes to C6 alcohols, and finally these alcohols are converted into the corresponding esters by alcohol acyltransferase enzymes [2]. C6 aldehydes and alcohols, and their derived esters, associated with green and fruity odor notes, are the key compounds in VOO aroma. Additionally, the contribution of compounds with five straight-chain carbons (C5 compounds), formed through an additional branch of the LOX pathway, to the aroma of VOO has also been proved [13]. Many compounds formed through the LOX pathway, globally known as oxylipins, exhibit strong biological activities and play important roles in the defense mechanisms of plants, both activating and regulating various defense responses by inducing genes related to other key biochemical pathways, and also in the immediate response to pathogens attacks through the antimicrobial activity of C6-aldehydes and esters [14,15].

The phenolic composition of VOO is directly related to the content of phenolic glucosides initially present in the olive fruit, such as the secoiridoids oleuropein, ligstroside, and demethyloleuropein, whose contents are genetically determined but modulated by environmental and physiological factors, or as a consequence of biotic and abiotic stresses [16]. The phenolic glucosides of the olive fruit are substrates of different enzymes during the oil extraction process [17]. The key enzymes acting on olive phenolic glucosides are β-glucosidases that hydrolyze these glucosides [18], forming secoiridoid aglucones containing the phenolic alcohols tyrosol (*p*-HPEA) and hydroxytyrosol (3,4-DHPEA), and the oxidoreductases, mainly polyphenol oxidase (PPO), which catalyzes the oxidative degradation of phenolic compounds and reduces the final phenolic content of VOO [19]. The characteristic phenolic profile of VOO comprises of five major groups of compounds: tyrosol and hydroxytyrosol containing compounds, lignans, flavonoids, and simple phenolic acids. Tyrosol and hydroxytyrosol derivatives, having a secoiridoid chemical structure, are the most abundant phenolic components in most olive oils. Among them, the aglucones of oleuropein (3,4-DHPEA-EA), ligstroside (*p*-HPEA-EA), decarboxymethyloleuropein (3,4-DHPEA-EDA, oleacein), and decarboxymethylligstroside (*p*-HPEA-EDA, oleocanthal) are those having the most prominent biological activities. As in the case of volatile compounds formed through the LOX pathway, some secoiridoid compounds act as defense chemicals against herbivores and pathogens. Similarly, the main enzymes involved in shaping the phenolic profile of the VOO, such as β-glucosidase and PPO, seem also to be associated with the plant’s natural defense mechanisms [20,21].

The two main biotic stressors of the olive fruit are the olive fruit fly, *Bactrocera oleae*, and the *Verticillium dahliae* fungus [22,23]. *B. oleae* is a dipteran of the *Tephritidae* family whose larvae feed on the pulp of the olive fruit causing multiple detrimental effects, such as the premature fall of the fruits, the decrease in oil yield, and/or negative alterations in the organoleptic properties of VOO [23]. The differences in susceptibility/tolerance of the different olive cultivars to the *B. oleae* pest may rely on mechanical factors (e.g., cuticle waxes), chemical factors (e.g., phenolic or volatile compounds), morphology of the fruits (e.g., size of the fruit), or a combination of them, so it is clear that a number of genes may be affected as a consequence of the infestation [24]. We have previously found that infection of Picual olive trees by the defoliating pathotype of *V. dahliae*, apart from reducing olive fruit yield, altered the balance between C6 and C5 volatile compounds in oil aroma while reducing the synthesis of the main phenolic compounds [25]. There is very little data on the impact of *B. oleae* infestation on the volatile profile of VOO and the few studies carried out on the effect of this pest on the phenolic composition of oils reported very different and sometimes contradictory results.

The aim of the present study was to analyze the changes induced by *B. oleae* infestation in the biosynthesis of volatile and phenolic compounds in the olive fruit (cvs. Picual, Manzanilla and Hojiblanca), and evaluate the effect of those changes on the deduced organoleptic and functional quality of VOO.

## 2. Results and Discussion

### 2.1. Effect of B. oleae Infestation on the Volatile Profile of VOO

**Figure 1:**
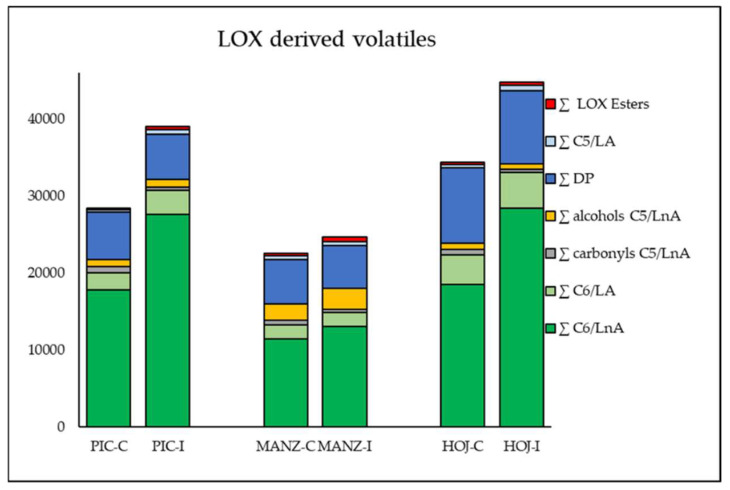
Content (ng g^−1^ oil) of the main groups of volatile compounds in oils extracted from infested (I) and non-infested (C) olive fruits (cvs. Picual, Manzanilla, and Hojiblanca). Data are mean contents from three different analyses.

Olive fruit infestation by *B. oleae* affects the volatile profile of the oils of Picual, Manzanilla, and Hojiblanca cultivars (Figure 1), producing significant changes both in the main families of volatile components of VOO (Table S1) and in some individual compounds with very different chemical/biochemical origins influencing the VOO aroma (Table S2). It is well known that oils from different olive genotypes exhibit a high level of variability regarding their volatile profiles [26]. Thus, the oils obtained from sound, non-infested, olive fruits from the three cultivars studied showed very different contents of C6 and C5 compounds (Figure 1). Hojiblanca oils had the highest content in C6 aldehydes derived from LNA (aldehydes C6/LnA) and LA (aldehydes C6/LA), as well as of LOX-derived esters, whereas Picual oils exhibited the highest content of C5 carbonyls derived from LA (carbonyls C5/LnA), and Manzanilla oils showed the highest contents of C5 alcohols derived from the same acid (alcohols C5/LnA). Despite these cultivar differences, a common pattern was observed in the volatile profiles of the oils obtained from *B. oleae* infested fruits. Thus, a statistically significant increase (*p* ≤ 0.05) in the content of C6/LnA was observed in the three cultivars, the most important group of volatile compounds produced through the LOX pathway (Supplementary Table S2). More specifically, the rise observed in the content of C6/LnA compounds of Manzanilla oils obtained from *B. oleae* infested fruits was around 15%, while the increments observed in Picual and Hojiblanca were around 50%. These data support previous findings on the increase of C6 volatiles as a consequence of plant–insect interaction [27].

In the three cultivars, the increases in the content of C6/LnA compounds were mainly due to the strong increase observed in the production of (*E*)-hex-2-enal, the most qualitatively and quantitatively relevant compound for VOO aroma due to its high concentration in most olive oils and its relatively low odor threshold [25,26]. Thus, the content of (*E*)-hex-2-enal in oils produced from *B. oleae*-infested Manzanilla fruits was double that of control oils, while the concentration of this compound in oils from infested Picual and Hojiblanca fruits tripled that of the control oils (Table S3). These results agree with those reported by Alagna et al. [11], who found that olive fruits from cvs. Leccino and Coratina infested with *B. oleae* larvae emitted higher amounts C6 aldehydes. These authors also observed the increase in the content of other volatile compounds, such as nonanal, acetic acid, and ethanol. However, we did not find significant increases in the content of nonanal in oils from infested fruits. The presence of this compound in VOO is exclusively associated with chemical oxidation processes, since its formation in olives cannot be linked to the LOX pathway, as it occurs in other fruits, given that olive HPL is strictly specific for 13-hydroperoxides and nonanal is formed by cleavage of 9-hydroperoxides [14]. We also observed a similar increase in the content of (*E*)-hex-2-enal after inoculation of Picual olive trees with the fungus *V. dahliae*, going from 66% of the content of C6/LnA compounds in control oils to 80% in oils from infested trees [25]. On the contrary, the pathogen *V. dahliae* caused a significant decrease in the content of C5/LnA compounds that we have not found in oils produced from fruits attacked by *B. oleae*-infested fruits. These differences could be explained by the induction of specific and different defense systems against pathogens and insects. Although there were no significant differences in terms of total content of C5/LnA compounds, a very significant decrease was observed in the content of carbonyls C5/LnA, which is mainly due to a strong decrease in the content of pent-1-en-3-one. Unlike the pentene dimers, which are the most abundant components of this group of compounds but have no impact on the aroma of the oil [26], pent-1-en-3-one has been described as an undesirable contributor to VOO aroma, providing green-pungent odor notes [13]. The content of this compound measured in oils from fruits attacked by the olive fruit fly is clearly lower than in control oils, 70, 35, and 50% in cvs. Picual, Manzanilla, and Hojiblanca, respectively. These data are also similar to those reported for *V. dahliae* infection in which the average content of this compound was 50% lower in oils from *V. dahliae* inoculated olives. Similarly, to what is found in oils from *V. dahlia*-infected olives, the content of LOX esters, the main contributors to oil fruitiness [26], was also significantly increased in the oils from *B. oleae*-infested fruits (Figure 1 and Table S2). To date, there are very few studies on the modification of the aroma of VOO due to *B. oleae* infestation, and those that exist have reported contradictory results, most likely because the oils were obtained from cultivars with different stages of maturation, cultivation areas, and extraction technologies [28,29]. In our study, these variables were fixed; thus, the three olive cvs. Picual, Manzanilla, and Hojiblanca were grown, harvested, and processed under identical conditions, showing a very similar response pattern to *B. oleae* infection in terms of green volatiles formed through the LOX pathway.

Recently, compounds with a different biochemical origin such as toluene, β-myrcene, limonene, and β-ocimene have been also linked to the infestation status of olive fruits attacked by *B. oleae* [30,31]. In this sense, we have also found significant increases in the content of ethanol, ethyl acetate, and β-ocimene in oils from *B. oleae*-infested fruits. Although ethanol and ethyl acetate are volatile components that appear in trace amounts in most oils [32], in our study, we have found significantly higher amounts of both compounds in the oils from infested fruits (Table S2). These increases would be related to fermentative processes triggered in the wounds caused by the fly. Ethanol and ethyl acetate are major contributors to the ‘fusty’ oil organoleptic defect, which is the most frequently identified by sensory evaluation in oils obtained from *B. oleae*-infested olive fruits [33]. Especially striking were the increases observed in the content of β-ocimene, a minor terpene compound, whose content significantly increased due to infestation, especially in oils from Picual and Manzanilla cultivars, where its concentration increased five and four times, respectively. According to Giunti et al. [31], β-ocimene is the volatile compound most positively correlated with the infestation of olive fruits by *B. oleae*. Accordingly, a high-moderate correlation (*r* = 0.626) was found for β-ocimene content in VOO and *B. oleae* infestation (Table S3). However, higher significant (*p* ≤ 0.05) positive and negative correlations were found for other volatile compounds and *B. oleae* infestation (Tables S2 and S3). Thus, the highest positive correlation coefficients were found for ethyl acetate (*r* = 0.822), (*E*)-hex-2-enal (*r* = 0.753), and (*E*)-hex-2-en-1-yl acetate (*r* = 0.764), while the most negative correlations were found for (*Z*)-hex-3-enal (*r* = −0.910), pent-1-en-3-one (*r* = −0.902), and (*Z*)-pent-2-enal (*r* = −0.927).

**Figure 2:**
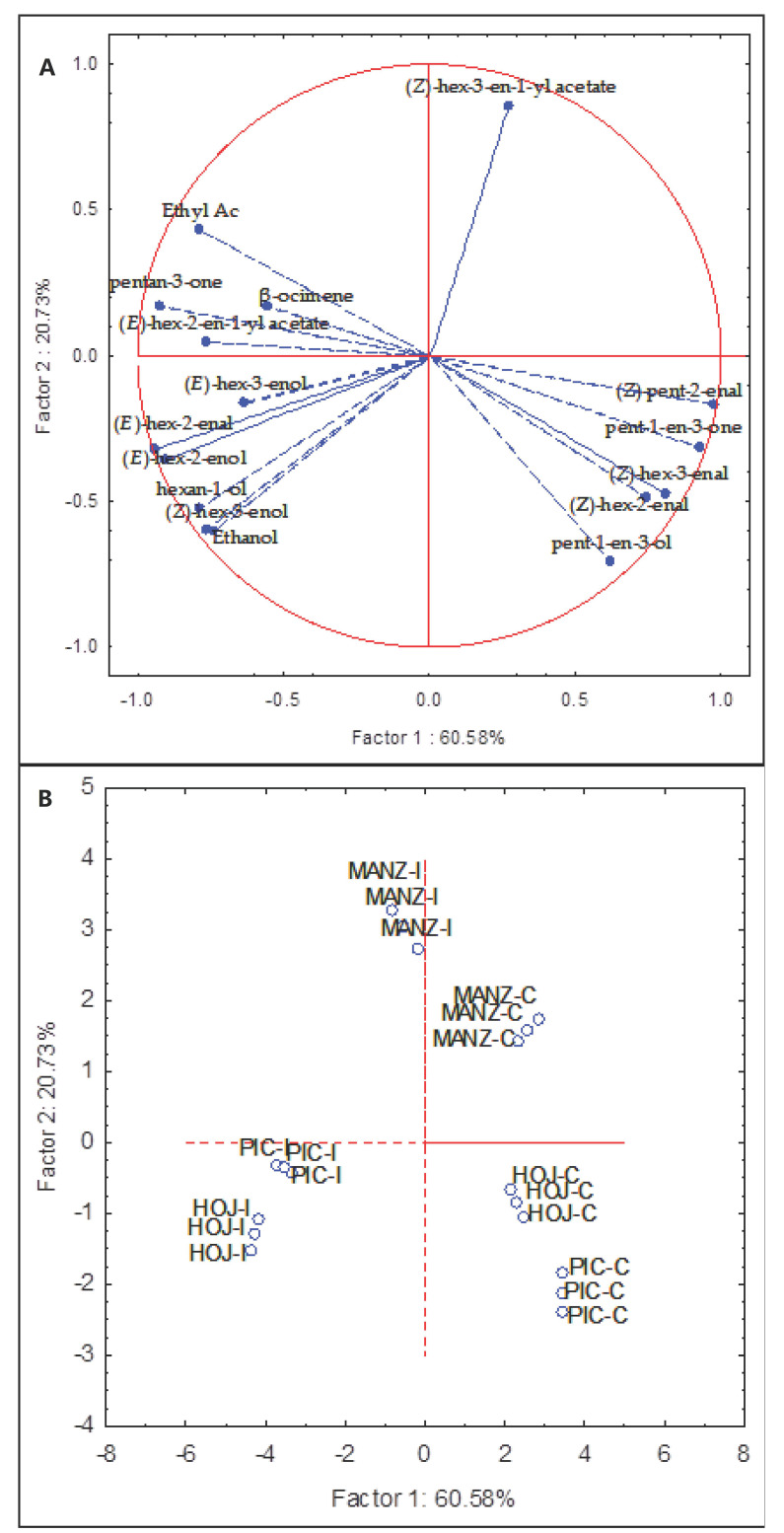
Principal component analysis of the main volatile components of oils from non-infested control (C) and infested (I) olive fruits (cvs. Picual, Manzanilla, and Hojiblanca). (**A**) vector distribution of the volatile compounds. (**B**) distribution of cultivars-infestation status.

PC analysis was applied using as variables the content of those volatile compounds with significant correlation coefficients (Figure 2). The first and second principal components described 80% of the total variability (PC1 60.58% and PC2 20.73%). PC1 was mainly explained in the positive part of the axis by the contents of pent-1-en-3-one and (Z)-pent-2-enal, while (E)-hex-2-enal is the major contributor to PC1 in the opposite sense (Figure 2A). PC2 positive part of the axis was mainly associated with the content of (Z)-hex-3-en-1-yl acetate and the contents of (*E*)-hex-3-enal and (*Z*)-hex-3-enal were the main contributors to PC2 negative part. Oils from uninfected fruits were distributed on the positive side of PC1, right part of the plot (Figure 2B), cv. Manzanilla in the upper quadrant and cvs. Picual and Hojiblanca in the lower one, while the oils from infested fruits were symmetrically located along the negative PC1 axis, left side of the plot.

### 2.2. Effect of Bactrocera oleae Infestation on the Phenolic Profile of VOO

**Figure 3:**
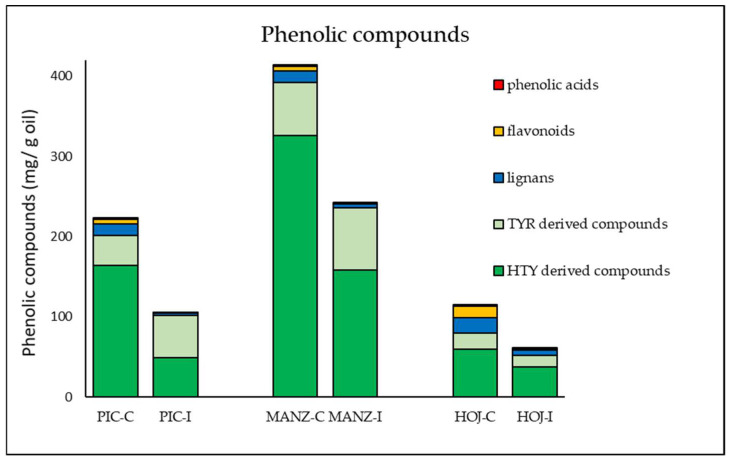
Content (µg g^−1^ oil) of the main groups of phenolic compounds in oils extracted from non-infested (C) and infested (I) olive fruits (cvs. Picual, Manzanilla, and Hojiblanca). Data are mean contents from three different analyses.

Figure 3 shows the content of the main groups of phenolic compounds in the oils obtained from non-infested (control) and *B. oleae*-infested fruits. Comparison of the phenolic composition of the control oils of the cultivars under study reveals evident cultivar differences according to the high variability of olive in terms of phenolic composition [34]. Among the cultivars analyzed in this study, cv. Hojiblanca showed the lowest phenolic content, while cv. Manzanilla exhibited the highest phenolic content, above 400 mg g^−^^1^ oil (Table 1). The most abundant compound in the three olive cultivars was 3,4-DHPEA-EA, which is associated with the bitter taste notes of VOO [35].

Despite the singularities of each cultivar, the total phenolic content was reduced by around 50% in the oils obtained from olive fruits infested with *B. oleae*. Although no conclusive results were found in some previous studies on the effect of *B. oleae* infestation on the VOO phenolic profile due to the great variability in the phenolic composition associated to other factors such as olive cultivar, growing practices, or extraction methods [36], significant correlations have been reported by some authors [37]. Thus, Koprivnjak et al. [33] found a negative linear correlation between the total phenolic content of oils from autochthonous Istrian olive cultivars and the infestation level of the fruits. Similar results were recently reported by Valencic et al. [38] when comparing the effect of active and damaging infestation in *cv.* Istrska belica fruits affected by *B. oleae*. These authors found very significant decreases in hydroxytyrosol derived compounds as did Gucci et al. in cv. Frantoio fruits with different degrees of damage [39]. Similarly, we have observed that the contents of hydroxytyrosol derivatives are those that experience the greatest decreases as a consequence of *B. oleae* infestation (Table 1), the decrease of 3,4-DHPEA-EA being especially notable, with reductions of 90%, 75%, and 70% in cvs. Picual, Manzanilla, and Hojiblanca, respectively. On the contrary, the secoiridoid derivatives containing tyrosol suffered only slight decreases or even notable increases as in the case of *p*-HPEA-EA in cvs. Picual and Manzanilla. Interestingly, Valencic et al. [38] also reported that compounds derived from tyrosol, such as *p*-HPEA-EA, increased their content in cv. Istrska belica oils proportionally to the severity of the infestation. On the contrary, we previously found in *V. dahliae*-infected Picual olives that the average content of both the tyrosol and the hydroxytyrosol derivatives were around 20% lower than in the control oils [25]. As previously mentioned in Section 2.1 in relation to the different effect of the pathogenic fungus and *B. oleae*, the differences observed in terms of phenolic compounds could also be explained by the existence of different defense systems against both biotic stressor, pathogen, and pest.

**Figure 4:**
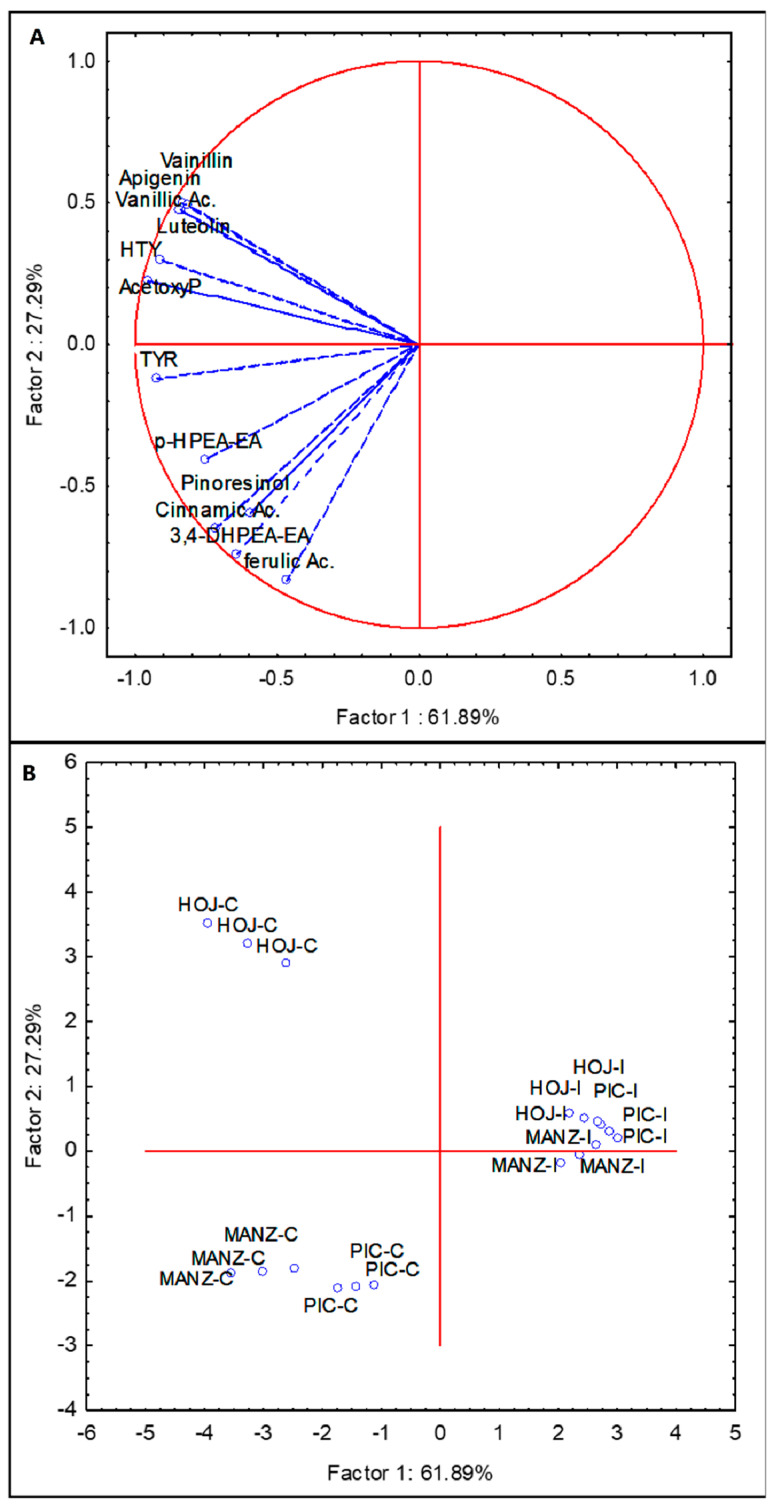
Principal component analysis of the main phenolic components of oils from non-infested (C) and infested (I) olive fruits (Picual, Manzanilla, and Hojiblanca). (**A**) vector distribution of the phenolic compounds. (**B**) distribution of cultivars-infestation status.

In order to explore the connection between VOO phenolics and *B. oleae* infestation, Pearson’s correlation coefficients were computed using the phenolic data of the oils from the three olive cultivars (Table S4). Significant negative correlation coefficients (*p* ≤ 0.05) were found for most phenolic compounds. Thus, content of acetoxypinoresinol showed the highest negative correlation coefficient with the fruit infestation (r = −0.91), followed by tyrosol and hydroxytyrosol. The content of 3,4-DHPEA-EA displayed the highest coefficient among the secoiridoid compounds (r = −0.69). PCA analysis was applied to the same subset of phenolic compounds with statistically significant correlation coefficients (Table S3). The first and second principal components described 89% of the total variability (PC1 61.89% and PC2 27.29%). The PCA bi-plot shows the great influence of BO infestation on the phenolic profile of VOO (Figure 4).

All control oils were clearly segregated and located in the left part of the plot (Figure 4B). The oils from cv. Hojiblanca were located in the upper quadrant, associated to the flavones lutein and apigenin, which are more abundant in this olive cultivar. The oils from cvs. Picual and Manzanilla, having a quite similar phenolic distribution, were located in the lower left quadrant, associated to *p*-HPEA-EA and 3,4-DHPEA-EA (Figure 4A). Despite the obvious cultivar differences reflected in the left part of the quadrant, after the fly attack, the oils show very similar phenolic profiles that do not segregate the different cultivars, as shown in the right part of the plot (Figure 4B).

As mentioned in the introductory section, it is generally accepted that the content of phenolic glucosides in olive fruit is the main factor determining the phenolic content of VOO. Accordingly, the lower phenolic content found in the oils of fruits infested by *B. oleae* could be due to a lower phenolic content in these fruits. However, the analysis of secoiridoid glucosides of the three olive cultivars in this study did not show significant differences between the phenolic content of healthy and infested Picual and Hojiblanca fruits, and even a significantly higher oleuropein and ligstroside contents were found in *B. oleae*-infested Manzanilla fruits compared to healthy fruits of the same cultivar (Table S5). The fact that the phenolic profiles of the fruits were not affected by the infestation but the content of phenolic compounds of the oils were drastically reduced suggests that *B. oleae* infestation could alter the status of the hydrolytic and oxidative enzymatic activities acting during the olive oil extraction process. Thus, the reduction of olive β-glucosidase activity (the key phenolic forming enzyme) and/or the increase in olive PPO (the main oxidative enzyme) would cause a decrease in the final phenolic content of the oils. The involvement of PPO in the reduction of the phenolic content of the oils associated to *B. oleae* infection was already suggested by Koprivnjaket al. [33] to explain the loss of phenolic content in oils obtained from infested olive fruits.

### 2.3. Effect of Bactrocera oleae Infestation on Genes Controlling the Biosynthesis of Volatile and Phenolic Compounds

A previous transcriptomic and proteomic study to investigate the molecular factors involved in infestation of cv. Moraiolo olives by *B. oleae* [40] found a substantial number of ESTs differentially expressed that were linked to wounding and to plant defense against biotic stress and abiotic stresses, as well as an important number of unidentified genes. More specifically, they found a remarkable enrichment of genes and proteins of the jasmonate signal transduction (LOX pathway) or phenylpropanoid metabolism pathways, both of which have as final products volatile and phenolic compounds directly involved in the chemical defense against pathogens. Great gene expression differences were also found by Grasso et al. [24] between resistant and susceptible olive cultivars, suggesting the implication of a great number of metabolites and signaling pathways in response to *B. oleae* infestation and pointing out the key role of some genes, such as those encoding β-glucosidases, in the defense system of the olive against this pest. Oleuropein activation by β-glucosidase is a well-known defense mechanism against herbivores. In fact, *B. oleae* requires a complex transcriptomic response in collaboration with its symbiont *Candidatus Erwinia dacicola* in order to infect and feed on olive fruits with high phenolic content [41].

With this background, the level of expression of key genes within the biosynthesis pathways of volatile and phenolic compounds, such as LOX and β-glucosidase, was studied in cv. Picual. Both gene families had been previously studied in our research group [14,42,43]. Among the *LOX* genes encoding proteins with 13-LOX activity, previous biochemical and gene expression data suggest the involvement of *Oep2LOX2* and to a lesser extent *Oep1LOX2* in the biosynthesis of VOO aroma. Similarly, previous biochemical and molecular characterization studies also point to the key role of *OeBGLU1A* and *OeBGLU1B* in the hydrolysis of phenolic glucosides of olive fruit that generate the main phenolic derivatives of VOO. Besides, based on the results obtained in the analysis of phenolic compounds in fruits and oils, we decided to study the possible involvement of *PPO* genes in relation to the effect of *B. oleae* infestation as well. Although different authors have reported the possible implication of PPO in the oxidation of phenolic compounds in olive, and its potential implication in the oxidative degradation of these compounds during the VOO production process, to date no PPO gene has been characterized in olive. Two *PPO* genes, *OePPO1* (GenBank accession number MW038828) and *OePPO2* (GenBank accession number MW038829), putatively involved in the oxidative degradation of olive phenolic compounds, were identified in an olive transcriptome previously generated [44]. Both genes were synthesized, cloned, expressed in *E. coli*, and purified as described in the Material and Methods section. Functional characterization studies carried out with natural phenolic substrates from olive fruit and VOO showed that both recombinant proteins were active against orthodiphenols, oleuropein, and hydroxytyrosol, but not to monophenols, such as tyrosol (data not shown). These data are in good agreement with the results previously obtained in in vitro oxidation experiments in which the incubations of VOO phenolic extracts with partially purified olive PPO from cv. Picual fruits showed a high oxidative degradation of the secoiridoid orthodiphenols 3,4-DHPEA-EDA, and 3,4-DHPEA-EA, but practically no alteration of the secoiridoid monophenols [19].

**Figure 5:**
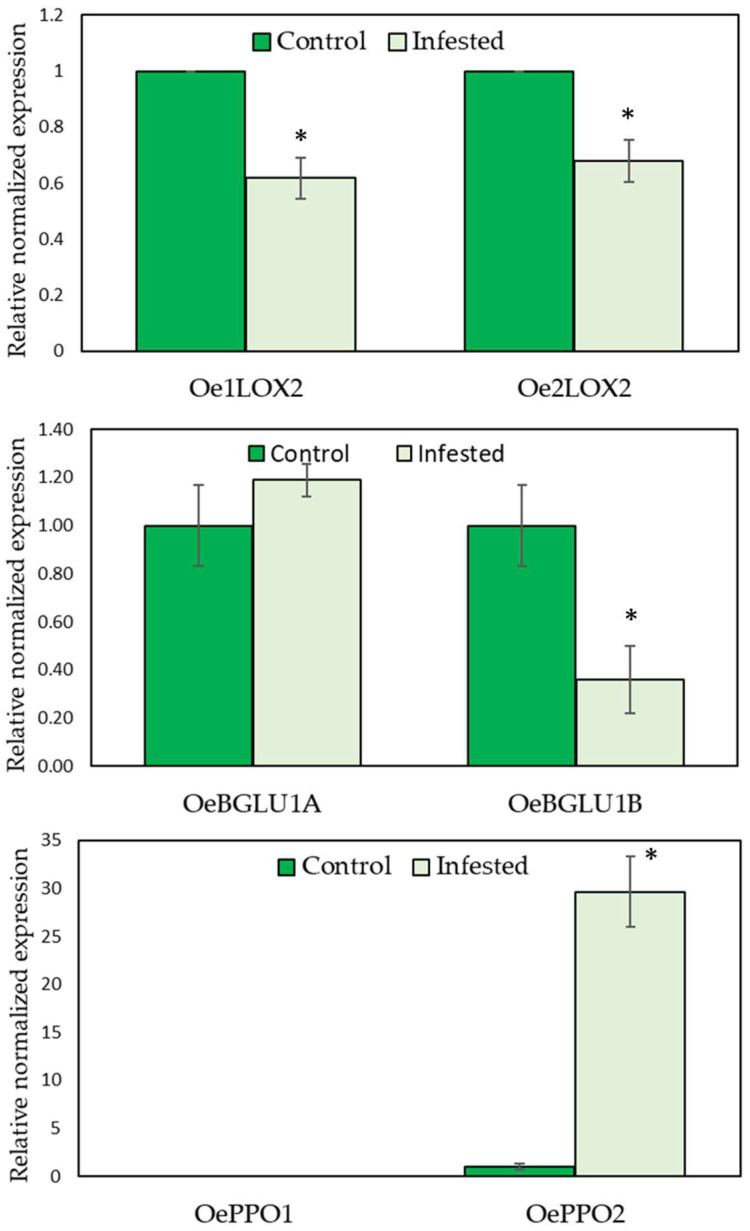
Relative expression levels of olive lipoxygenase (*Oep1LOX2*, *Oep2LOX2*), β-glucosidase (*OeBGLU1A*, *OeBGLU1B*) and PPO (*OePPO2*, *OePPO2*) olive genes in the mesocarp tissue of olive fruits (cv. Picual). Data are mean ± SD. Three biological and two technical replicates were obtained for each sample. (*) Indicate statistically significant differences according to Tukey’s test (*p* ≤ 0.05).

The changes induced by *B. oleae* infestation in the relative expression levels of the selected LOX, β-glucosidase, and PPO olive genes were analyzed by RT-QPCR (Figure 5). The two selected LOX genes have 13-LOX activity and therefore produce 13-hydroperoxides that are later transformed by 13-HPL into C6 aldehydes and subsequently into alcohols and volatile esters that determine the green aroma notes of VOO [14]. The data we have on the regulation of their expression suggest that *Oep2LOX2* is mainly responsible for the biosynthesis of the VOO aroma, while *Oep1LOX2* is more related to the response to wounding, and probably focused on the synthesis of jasmonic acid. No significant differences were found in the expression level of either of the two *LOX* genes in olive fruits infested by *B. oleae* (Figure 5). These results apparently do not agree with the highest concentration of volatile compounds derived from LOX pathway found in oils obtained from infested fruits, especially in terms of (*E*)-hex-2-enal content. However, we have previously observed that, although the expression of *Oep2LOX2* is not affected, the expression of *Oep1LOX2* is rapidly but transiently induced as a consequence of mechanical damage in the olive fruit, reaching its maximum level 90 min after wounding [14]. This brief induction of gene expression caused an increase in LOX activity, sustained over time, which could explain the increase in the biosynthesis of C6 compounds during oil extraction from infested fruits despite not detecting a higher expression levels of the *Oep1LOX2* gene.

Regarding the metabolism of phenolic compounds, the two β-glucosidase genes directly involved in the formation of the secoiridoid derivatives present in VOO did not show a significant modification of their expression that could explain the lower content of phenols found in the oils obtained from *B. oleae*-infested fruits (Figure 5). The expression of *OeBGLU1A*, whose product has a powerful hydrolytic activity on oleuropein and is mainly responsible for the hydrolysis of phenolic glucosides during the oil extraction process [42], experimented a slight but not statistically significant increase, which seems to support previous findings on the active role of β-glucosidase in the olive defense system [42]. The parallel decrease of *OeBGLU1B*, whose molecular characteristics suggest that it could also be involved in the synthesis of VOO secoiridoids, although with a very modest participation according to its very low expression level [43], does not justify the drastic decrease observed in the content of phenolic compounds in the oils from infested fruits.

Unlike β-glucosidase, the study of PPO gene expression levels did show very significant differences between control fruits and fruits infested by *B. oleae*. The expression of *OePPO1* showed a negligible level in both groups of fruits, control and infested (Figure 5), in line with the level that we had previously found in different olive cultivars, including *cv.* Picual [44]. However, a strong induction of *OePPO2* expression, which has a significantly higher mean expression level in the olive transcriptome [44], was observed as a consequence of *B. oleae* infestation, 30 times higher in infested fruits than in the control (Figure 5). The effects of PPO-mediated oxidative defenses against different herbivore pathogens have been reported in plants such as tomato, tea, or strawberry [21]; however, the specific mechanisms are largely unknown. Koprivnjak et al. [33] already proposed that the increase in endogenous PPO activity in olive fruits, together with the greater exposure to oxygen due to cell breakage and damage associated to the wound caused by the fly, could explain the notable decrease that they observed in the phenolic content of oils obtained from *B. oleae* infested fruits from two Slovenian olive cultivars. The data obtained in our study are the first evidences that would support this hypothesis. According to the results, the level of PPO enzymatic activity of the fruits would increase enormously as a consequence of the strong induction of the *OePPO1* gene in fruits infested by *B. oleae*, which in turn would lead to an increase in the oxidative degradation of phenolic compounds during the oil extraction process, despite the fact that both the phenolic glucoside content and the level of β-glucosidase activity of the fruit remain unchanged after infestation.

## 3. Materials and Methods

### 3.1. Plant Material

Three Spanish olive cultivars (*Olea europaea* L.) were selected (Manzanilla, Picual, and Hojiblanca), which, based on their harvesting dates and fruit sizes, are quite susceptible to *B. oleae* pest. Olive trees were grown in the same agroclimatic conditions at the experimental orchards of Instituto de la Grasa (Sevilla, Spain), 6 × 5 m spacing, using irrigation supply by in-line drippers to avoid water stress of plants (290 mm, Mars-October), containing N (80 kg/year/ha), P2O5 (40 kg/year/ha), and K2O (110 kg/year/ha). Each olive cultivar was harvested at the optimum commercial maturity when most fruits were at turning color. Fruits with total green or black skin color were discarded. Manzanilla fruits were harvested at mid-October and Picual and Hojiblanca at mid-November. Around 10 kg of fruits of each cultivar were handpicked from five different trees, immediately transported to the laboratory and divided into two groups, one of apparently healthy fruits, with no symptoms of fly attack, and another group of fruits with obvious symptoms of severe olive fruit fly attack (fruits with exit holes).

### 3.2. Olive Oil Extraction

Olive oil was extracted using an Abencor analyzer (Comercial Abengoa, S.A., Seville, Spain) that simulates the industrial process of VOO production on a laboratory scale. Processing parameters have been described in a previous paper [18].

### 3.3. Extraction and Analysis of Fruit and VOO Phenolic Compounds

Fruit phenolic compounds were extracted according to a recently developed protocol [45]. Mesocarp tissue slices were cut and kept at 4 °C for 72 h in dimethyl sulfoxide (6 mL/g of fruit), containing syringic acid (24 mg/mL) as an internal standard. The extracts were filtered through a 0.45 μm mesh nylon and kept at −20 °C until HPLC analysis.

VOO phenolic compounds were isolated by solid phase extraction (SPE) on a diol-bonded phase cartridge (Supelco, Bellefonte, PA, USA) based on the method by Mateos et al. [46] using *p*-hydroxyphenylacetic and *o*-coumaric acids as internal standards.

Phenolic compounds from fruits and oils were analyzed (three analyses per sample) by HPLC on a Beckman Coulter liquid chromatography system (Beckman, Fullerton, CA, USA) composed of two modules a 168 detector, a solvent module 126, an autosampler module 508, and a Waters column heater module following a previously described methodology [47]. A Superspher RP 18 column (4.6 mm i.d. × 250 mm, particle size 4 µm: Dr Maisch GmbH, Ammerbuch, Germany) at flow rate 1 mL min^−1^ and a temperature of 35 °C were used for all the analyses. Tentative identification of compounds was carried out by their UV-Vis spectra and confirmed by HPLC/ESI-qTOF-HRMS on a liquid chromatograph Dionex Ultimate 3000 RS U-HPLC liquid chromatograph system (Thermo Fisher Scientific, Waltham, MA, USA) equipped with a similar column and using the same elution program. Mass spectra were acquired in MS fullscan mode and data were processed using Target Analysis 1.2 software (Bruker Daltonics, Bremen, Germany). The quantification of the phenolic components was done using response factors calculated for each phenolic compound.

### 3.4. Extraction and Analysis of VOO Volatile Compounds

Olive oil samples (0.5 g of oil in 10 mL vials) were placed in a vial heater at 40 °C and after a 10 min equilibration, volatile compounds were adsorbed onto a SPME fiber DVB/Carboxen/PDMS 50/30 μm (Supelco Co., Bellefonte, PA, USA) for 50 min. Desorption of volatile compounds trapped in the SPME fiber was performed directly into the GC injector. Identification of volatile compounds was carried out on a 7820A/GC-5975/MSD system (Agilent Technologies, Santa Clara, CA, USA) equipped with a DB-Wax capillary column (60 m × 0.25 mm i.d., film thickness, 0.25 μm, J&W Scientific, Folsom, CA, USA). Quantitation of VOO volatile compounds was carried out on a HP-6890 gas chromatography apparatus (Agilent Technologies, Santa Clara, CA, USA), equipped with the same column and operated under similar conditions (three analyses per sample). Details on the operating conditions used for both systems 7820A/GC-5975/MSD and HP-6890, as well as the quantification method have been fully described in a previous paper [48]. The volatile compounds were individually quantitated and also clustered into different groups and subgroups according to their origin in the LOX pathway branch (C6 and C5 compounds) from LA and LNA, as well as terpenes, and branched-chain (BC) volatile compounds from amino acid metabolism:

- C6/LnA aldehydes: (*E*)-hex-2-enal, (*Z*)-hex-3-enal, (*Z*)-hex-2-enal, (*E*)-hex-3-enal.
- C6/LnA alcohols: (*E*)-hex-2-enol, (*Z*)-hex-3-enol, (*E*)-hex-3-enol.
- C6/LA aldehyde: hexanal.
- C6/LA alcohol: hexan-1-ol.
- C5/LNA carbonyls: pent-1-en-3-one, (*E*)-pent-2-enal, (*Z*)-pent-2-enal.
- C5/LNA alcohols: pent-1-en-3-ol, (*E*)-pent-2-en-1-ol, (*Z*)-pent-2-en-1-ol.
- PD: pentene dimers (seven isomers).
- C5/LA carbonyls: pentan-3-one, pentanal.
- C5/LA alcohol: pentan-1-ol.
- LOX esters: hexyl acetate, (*Z*)-hex-3-en-1-yl acetate, (*E*)-hex-2-en-1-yl acetate.
- Non-LOX esters: methyl acetate, ethyl acetate, methyl hexanoate, ethyl hexanoate.
- Terpenes: limonene, β-ocimene
- BC aldehydes: 3-methyl-butanal, 2-methyl-butanal
- BC alcohol: 2-methyl-butan-1-ol

### 3.5. Identification of Polyphenol Oxidase Full-Length cDNA

Two putative PPO genes that might be involved in the oxidative degradation of olive phenolic compounds were selected based on the gene expression levels and the differential expression data from an olive transcriptome generated from seven olive cultivars [44] referenced to an olive genome database (OE6.OLIVEFAT, https://denovo.cnag.cat/olive_data; accessed on 27 November 2020) for annotation.

### 3.6. OePPO Genes Cloning, Heterologous Protein Expression, Purification, and Functional Characterization

The coding sequences of the selected candidate genes, *OePPO1* (OE6A068152) and *OePPO2*, (OE6A114203) were synthesized with *E. coli* codon optimization (GenScript, Piscataway, NJ, USA) and cloned into a pGEX6P.1 vector as EcoRI-XhoI fragments. Two constructs were obtained to be produced as glutathione-S-transferase (GST) fusion proteins: GST-OePPO1 and GST-OePPO2.

Protein expression and purification were carried out according to Kampatsikas et al. [49] with minor modifications. BL21(DE3) lacIq E. coli cells containing OePPO1 or OePPO2 constructs were grown at 37 °C in Luria Bertani media (LB) with 0.5 M NaCl to OD_600_ of 0.6, then supplemented with 0.5 mM isopropyl-b-D-thiogalactoside (IPTG) and 0.5 mM CuS and grown for 20 h at 19 °C. Cells were harvested by centrifugation, resuspended in lysis buffer (50 mM Tris-HCl, pH 7.5, 0.2 M NaCl, 1 mM EDTA, glycerol 10% (*v/v*), 1 mM benzamidine, 1 mM phenylmethanesulfonyl fluoride), and lysed by sonication. The resulting crude protein lysate was clarified by centrifugation prior to chromatographic purification with Sepharose-GSH beads (GE Healthcare, Chicago, IL, USA) following the manufacturer’s instructions. The soluble fraction obtained was added to the beads for GST-OePPOs purification and incubated for 2 h at 4 °C in a 360 ° rotator. Then, recombinant protein-bound-beads were incubated in preScission Protease proteolytic digestion buffer (GE Healthcare) (50 mM Tris HCl pH 7.0, 150 mM NaCl, 1 mM EDTA) and 160 U/mL of protease. The enzymatic digestion was carried out overnight at 4 °C on a 360° rotator to eliminate the GST fusion protein from the OePPO1 and OePPO2 proteins. The purified proteins were collected as eluates after centrifugation. OePPO1 and OePPO2 purified protein buffer was exchanged to 100 mM Tris-maleate pH 6.8 and 10% glycerol buffer on a PD-10 column (Sephadex G-25, GE Healthcare, Chicago, IL, USA) and further concentrated using a Vivaspin centrifugal concentrator (MWCO 30 kDa, Merck, Darmstadt, Germany). Purity of the recombinant proteins was evaluated by SDS-PAGE and the protein concentration was determined by the Bradford assay [50].

The PPO enzyme assays for functional characterization of PPO recombinant proteins were performed in 150 µL of reaction buffer (50 mM Tris-maleate pH 6.8, 1 mM SDS) containing 10–20 µg of recombinant protein, using as substrates (2.5 mM) two olive phenolic components: hydroxytyrosol (Sigma-Aldrich, St. Louis, MO, USA) and oleuropein (Extrasynthese, Genay, France). The mixture was incubated at 25 °C for 5 min and then the reaction was terminated by the addition of 150 µL methanol. The reaction mixture was centrifuged and filtered (0.45 µm) and the supernatant was analyzed by HPLC using the same equipment and chromatographic conditions previously described for the analysis of phenolic compounds. In all cases, controls were carried out using the same reaction mixture, but carrying untransformed *E. coli* BL21 protein extracts.

### 3.7. Total RNA Extraction and Gene Expression Analysis

Full details of RNA extraction and gene expression analysis have been recently reported [43]. Total RNA extraction from olive mesocarp tissues was performed using the Spectrum Plant Total RNA Kit (Sigma-Aldrich, St. Louis, MO, USA) and cDNAs were synthesized using the Ready-To-Go T-Primed First Strand Kit (Amersham Bioscience, Roosendaal, The Netherlands). The cDNAs were subjected to RT-QPCR using SYBR Green I (SsoAdvancedTM Universal SYBR^®^ Green Supermix, BioRad) in a CFX96 Touch System (BioRad) to monitor the resulting fluorescence. Two olive genes, elongation factor-1-alpha (OeEF1α), and glyceraldehyde-3-phosphate dehydrogenase (OeGAPDH) (Olive genome cv Farga Data Base annotation number OE6A045598 and OE6A105640, respectively) were selected as reference genes according also to previous validation studies [43]. Specific pair of primers for these reference genes and genes under study are described in Table S6. Three biological and two technical replicates were obtained from each sample.

### 3.8. Statistical Analysis

Data were statistically evaluated using Statistica (Statsoft, Inc., Tulsa, OK, USA). Correlations among phenolic and volatile compounds analyzed in the oils and the infestation status of the olive fruits were analyzed using Pearson’s correlations. Principal component analysis (PCA) was used to evaluate the levels of association between the infestation and the phenolic and volatile compounds analyzed in the oils from the three olive cultivars selected. For gene expression analysis, statistical significance was set at a level of *p* ≤ 0.05 (Student’s *t*-test).

## 4. Conclusions

Despite cultivar differences, we found that BO infestation caused very similar effects on the aroma and flavor properties of VOOs produced from Manzanilla, Picual, and Hojiblanca olive fruits. The oils obtained from infested olive fruits showed significant increases in the content of the main C6 volatile compounds formed through the LOX pathway, with concentrations of (E)-hex-2-enal two or three times higher than those of the control oils, which corroborate the role of these volatile compounds in the defense mechanisms of the plant. Significant increases in the contents of ethanol, ethyl acetate, and β-ocimene were also associated to *B. oleae* infestation. On the contrary, *B. oleae* infestation caused a drastic decrease on the phenolic content of the oils. The expression analysis of key genes involved in the biosynthesis of volatile and phenolic components of VOO suggest that the olive possesses a PPO-mediated oxidative defense system similar to those described in other plants against herbivorous pathogens. In this way, we found that the infestation of olive fruits by *B. oleae* leads to a very strong induction of the OePPO2 gene, which codes for a very active protein in the oxidation of olive o-diphenols, which would explain the reduction in the phenolic content of the oils due to increased oxidative activity during the oil extraction process.

## Figures and Tables

**Table 1:** Phenolic compounds analyzed in virgin olive oils obtained from non-infested (C) and infested (I) olive fruits (cvs. Picual, Manzanilla, and Hojiblanca).

Compounds (mg/g Oil)	-					
	Picual-C	Picual-I	Manz-C	Manz-I	Hojib-C	Hojib-I
Hydroxytyrosol	0.6b *	0.2a	1.6b	0.3b	1.9b	0.4a
Tyrosoll	1.9b	0.4a	4.3b	0.8b	3.0b	0.7a
Vanillic acid	0.2b	0.1a	0.2b	0.1b	0.4b	0.1a
vanillin	0.1a	0.1a	0.1a	0.1b	0.2b	0.1a
p-coumaric acid	0.3a	0.5b	0.2a	0.1b	0.6b	0.2a
Hydroxytyrosol ac.	2.7a	5.9b	7.3a	13.7b	1.8a	2.6b
3,4-DHPEA-EDA	24.8a	28.7a	121.0b	96.7a	24.3a	24.9a
p-HPEA-EDA	22.7a	41.9b	44.5a	64.2b	3.6a	3.5a
Pinoresinol	3.2b	1.1a	2.2b	1.2b	1.6b	1.5a
Cinnamic acid	1.1b	0.1a	0.9b	0.1b	0.4b	0.2a
Acetoxipinoresinol	11.0b	1.5a	12.3b	3.3b	17.7b	4.9a
3,4-DHPEA-EA	135.6b	14.7a	196.3b	48.1b	31.8b	9.7a
p-HPEA-EA	13.3b	9.9a	16.9b	12.6b	12.9b	10.6a
Ferulic acid	0.2b	0.1a	0.2b	0.1b	0.0	0.0a
Luteolin	4.8b	0.7a	5.4b	0.6b	12.7b	1.4a
Apigenin	0.9b	0.1a	0.9b	0.2b	2.2b	0.3a
Total phenolics	223.3b	106.1a	414.4b	242.1b	115.2b	61.2a
o-diphenolics	168.5b	50.2a	331.6b	159.5b	72.5b	39.0a
Secoiridoids	196.3b	95.2a	378.7b	221.6b	72.7b	48.8a

(*) Different letters indicate statistically significant differences according to Tukey’s test (*p* ≤ 0.05).

## Data Availability

Not applicable.

## Supplementary Materials

The following supporting information can be downloaded online. Table S1: Groups of volatile compound (according to their biochemical origin) analyzed in the oils; Table S2: Individual volatile compounds analyzed in the oils; Table S3: Correlation coefficients between VOO volatile compounds and infestation status Table S4: Correlation coefficients between VOO phenolic compounds and infestation status; Table S5: Phenolic composition of olive fruits.; Table S6: PCR primers used.
