# Supplementary material for: The Infestation of Olive Fruits by Bactrocera oleae (Rossi) Modifies the Expression of Key Genes in the Biosynthesis of Volatile and Phenolic Compounds and Alters the Composition of Virgin Olive Oil
Source: Molecules. 2022 Mar 2;27(5):1650. doi: 10.3390/molecules27051650 (PMC8911628; doi:10.3390/molecules27051650)
Supplement: Supplementary file 1 [file molecules-27-01650-s001.zip › molecules-1592491-supplementary.pdf]

Supplementary Information

The Infestation of Olive Fruits by *Bactrocera oleae* (Rossi) Modifies the Expression of Key Genes in the Biosynthesis of Volatile and Phenolic Compounds and Alters the Composition of Virgin Olive Oil

Andrés Notario, Rosario Sánchez, Pilar Luaces, Carlos Sanz and Ana G. Pérez *

Table S1: Groups of volatile compound (according to their biochemical origin) analyzed in the oils.

	Manzanilla		Picual		Hojiblanca	
**Compounds (ng/g oil)**	**Control**	**Infested**	**Control**	**Infested**	**Control**	**Infested**
**∑ aldehydes C6/LnA**	10255,8a	11960,4b	15339,5a	22678,6b	16990,2a	22996,7b
**∑ alcohols C6/LnA**	1075,1a	1167,5a	2420,6a	4879,7b	1507,1a	5423,4b
**∑ C6/LnA**	11423,3a	13035,5b	17760,1a	27558,4b	18497,4a	28420,1b
**∑ aldehydes C6/LA**	1515,8a	1547,1a	1734,9a	2506,2b	3495,9a	3440,0a
**∑ alcohols C6/LA**	271,4a	282,5a	463,0a	698,8b	341,2a	1129,2b
**∑ C6/LA**	1787,3a	1829,6a	2197,9a	3205,0b	3837,1a	4569,2b
**∑ carbonyls C5/LnA**	614,6b	418,2a	878,9b	320,1a	687,0b	409,2a
**∑ alcohols C5/LnA**	2104,5a	2676,5b	868,7a	1036,2b	796,7b	747,0a
**∑ DP**	5835,1a	5544,7a	6146,9a	5846,7a	9846,9a	9461,8a
**∑ C5/LnA**	8544,3a	8639,5a	7894,6a	7203,0a	11330,8a	10618,1a
**∑ carbonyls C5/LA**	397,4a	527,3b	236,8a	529,2b	278,2a	658,6b
**∑ alcohols C5/LA**	66,7a	49,0a	103,1a	129,8a	85,0a	132,4b
**∑ C5/LA**	464,1a	576,4b	339,9a	659,1b	363,3a	791,0b
**∑ esters LOX**	324,0a	547,9b	215,1a	419,9b	369,7a	410,9b
**∑ esters no LOX**	1130,0b	600,9a	1028,6a	1490,7b	1108,2b	1002,7a
**∑ esters**	1454,1b	1148,8a	1243,7a	1910,7b	1477,9a	1413,6a
**∑ aldehydes AA**	53,2a	108,5b	14,0a	22,65b	45,6a	46,8a
**∑ alcohols AA**	3,5a	4,3a	9,1a	16,2b	3,8b	2,6a
**∑ volatiles AA**	56,8a	112,8b	23,1a	38,8b	49,4a	49,4a

**∑ terpenes**	11753,4b	10864,3a	9096,9a	9054,9a	751,7a	931,2b

(*) mean contents from three different analyses

(**) different letters within the same olive cultivar indicate statistically significant differences ($p \leq 0.05$)

Table S2

Compounds (ng/g oil)	Picual C			Picual I			Manzanilla C			Manzanilla I			Hojiblanca C			Hojiblanca I		
Ethyl acetate	251.3	±	6.3	568.1	±	14.9	448.4	±	10.1	721.1	±	56.4	502.6	±	14.0	728.9	±	0.3
Ethanol	504.4	±	3.3	912.6	±	32.2	231.4	±	20.9	341.9	±	9.8	767.0	±	54.9	1280.5	±	20.4
E3-hexenal	844.6	±	371.5	1729.6	±	57.5	613.4	±	40.6	283.6	±	15.4	627.3	±	21.0	461.1	±	0.1
Z3-hexenal	9229.6	±	202.8	5277.9	±	94.9	6963.9	±	66.0	3119.0	±	238.8	8946.3	±	538.6	3137.5	±	42.7
Z2-hexenal	776.0	±	24.2	478.0	±	30.6	676.1	±	50.3	332.2	±	13.5	945.3	±	56.3	437.1	±	4.3
E2-hexenal	4489.3	±	95.7	15193.1	±	247.5	3707.0	±	317.1	6521.0	±	214.0	6471.3	±	223.6	18961.0	±	190.9
E3-hexenol	20.6	±	3.5	103.9	±	1.3	10.4	±	0.4	24.4	±	1.0	26.0	±	0.4	31.9	±	1.2
Z3-hexenol	2153.9	±	2.4	3684.6	±	58.0	854.2	±	44.4	875.0	±	7.7	1234.3	±	19.7	3782.3	±	50.5
E2-hexenol	246.1	±	16.3	1091.3	±	42.9	210.6	±	40.2	268.2	±	0.8	246.8	±	4.2	1609.2	±	22.5
hexanal	1734.9	±	60.3	2506.2	±	20.6	1515.9	±	27.2	1547.1	±	58.4	3495.9	±	29.8	3440.1	±	46.3
hexanol	463.0	±	10.4	698.8	±	9.4	271.5	±	36.4	282.5	±	12.2	341.3	±	5.3	1129.2	±	11.1
1-penten-3-one	682.6	±	16.3	198.7	±	5.9	478.5	±	18.1	311.6	±	0.9	506.8	±	25.6	243.1	±	8.6
Z2-pentenal	80.8	±	3.5	23.3	±	0.7	62.7	±	3.7	41.1	±	0.0	69.1	±	3.8	21.0	±	0.5
E2-pentenal	115.6	±	6.4	98.1	±	5.9	73.5	±	8.0	65.5	±	3.9	111.2	±	2.3	145.1	±	5.0
1-penten-3-ol	181.8	±	13.7	126.1	±	6.5	135.0	±	9.8	120.6	±	5.2	158.1	±	6.5	137.1	±	7.5
Z2-pentenol	485.2	±	3.3	576.4	±	10.6	392.5	±	10.0	595.4	±	17.1	336.4	±	7.6	320.1	±	6.2
E2-pentenol	201.7	±	2.3	333.8	±	8.0	1577.0	±	43.9	1960.6	±	37.5	302.3	±	4.2	289.9	±	6.8
DP-1	278.6	±	8.3	252.3	±	6.1	240.5	±	12.7	248.3	±	12.5	501.9	±	34.8	466.2	±	16.1
DP-2	221.0	±	4.8	210.2	±	4.9	200.6	±	8.3	213.9	±	9.6	387.0	±	39.9	364.8	±	6.9
DP-3	1092.5	±	17.8	1105.8	±	0.3	1049.3	±	25.4	1136.7	±	56.3	2069.2	±	44.6	2070.9	±	41.7
DP-4	1985.3	±	40.1	1489.6	±	33.9	1687.7	±	111.2	1501.6	±	141.1	2525.3	±	135.3	2249.6	±	41.7
DP-5	317.0	±	22.0	339.5	±	3.0	365.1	±	9.0	403.0	±	14.1	510.2	±	17.1	568.9	±	19.2
DP-6	1290.8	±	40.5	1265.5	±	64.6	1114.2	±	107.6	1525.4	±	517.1	1740.3	±	245.6	2154.3	±	82.6
DP-7	961.7	±	371.0	1183.9	±	91.0	1177.7	±	320.0	515.9	±	515.9	2113.1	±	350.6	1587.1	±	506.9
2+3-pentanone	170.1	±	19.5	476.7	±	39.7	324.8	±	24.6	444.5	±	30.2	245.8	±	30.6	634.4	±	50.5
pentanal	66.7	±	3.6	52.5	±	2.7	72.6	±	5.0	82.8	±	0.8	32.5	±	10.6	24.2	±	0.5
pentanol	103.1	±	24.7	129.9	±	23.9	66.7	±	4.8	49.1	±	37.6	85.1	±	3.8	132.4	±	0.9
Hexy acetate	94.9	±	18.7	221.5	±	1.2	158.9	±	19.9	325.5	±	3.9	203.3	±	5.3	233.9	±	2.9
E2-hexenyl acetate	106.6	±	17.0	170.8	±	26.3	90.5	±	18.0	158.0	±	42.5	132.5	±	1.0	161.6	±	0.4
Z3-hexenyl acetate	13.6	±	2.0	27.7	±	9.0	74.7	±	25.4	64.4	±	5.2	33.9	±	6.2	15.4	±	2.7
Methyl acetate	41.3	±	3.5	59.6	±	4.9	2.6	±	0.2	17.3	±	0.3	7.6	±	1.0	22.8	±	1.1
Methyl hexanoate	27.2	±	1.2	29.4	±	0.8	99.6	±	3.1	98.1	±	1.5	55.3	±	1.8	39.3	±	1.9
Ethyl hexanoate	957.8	±	72.6	1398.6	±	96.6	1027.1	±	5.6	483.7	±	25.6	1045.0	±	37.3	939.5	±	296.0
2-Me-butanal	9.5	±	0.9	11.6	±	2.2	41.5	±	5.9	83.0	±	9.6	20.3	±	6.4	24.2	±	1.6
3-Me-butanal	4.5	±	0.4	11.0	±	0.5	11.8	±	2.4	25.5	±	3.7	25.4	±	6.3	22.6	±	0.8
2+3-Methyl-butanol	9.2	±	0.8	16.2	±	2.1	3.6	±	0.4	4.3	±	0.4	3.9	±	0.2	2.6	±	0.2
limonene	13.2	±	13.2	8.2	±	0.4	19.0	±	6.6	13.3	±	2.0	104.9	±	3.8	211.9	±	1.9
ß-Ocymene	1921.9	±	398.6	10369.9	±	487.2	1272.5	±	31.2	4881.9	±	101.5	1594.6	±	94.7	1709.7	±	2.1

Table S3. Significant Pearson correlation coefficients (p≤0.05) between the infestation status and the main volatile compounds found in VOO.

Compound	*r*
ethyl acetate	0.822
ethanol	0.483
(*Z*)-hex-3-enal	-0.910
(*Z*)-hex-2-enal	-0.897
(*E*)-hex-2-enal	0.753
(*E*)-hex-3-enol	0.554
(*Z*)-hex-3-enol	0.553
(*E*)-hex-2-enol	0.694
hexan-1-ol	0.566
pent-1-en-3-one	-0.902
(*Z*)-pent-2-enal	-0.927
pent-1-en-3-ol	-0.688
pentan-3-one	0.864
(*E*)-hex-2-en-1-yl acetate	0.764
(*Z*)-hex-3-en-1-yl acetate	-0.096
β-ocimene	0.626

Table S4: Statistically significant correlation coefficients between VOO phenolic compounds and infestation by BO

Compounds (ng/g oil)	Correlation Coefficient
Hydroxytyrosol	-0.802598
Tyrosoll	-0.856506
Vanillic acid	-0.787263
vanillin	-0.728496
Pinoresinol	-0.744461
Cinnamic acid	-0.831550
Acetoxipinoresinol	-0.915263
3,4-DHPEA-EA	-0.699919
p-HPEA-EA	-0.696055
Ferulic acid	-0.579958
Luteolin	-0.754357
Apigenin	-0.731996

Table S5. Phenolic composition of olive fruits

	**Manzanilla**		**Picual**		**Hojiblanca**	
**Phenolic compounds (μg/g FW)**	**Control**	**Infested**	**Control**	**Infested**	**Control**	**Infested**
**Hydroxytyrosol glucoside**	3119,9b	1870,8a	781,6a	1091,5b	3172,5b	2776,1a
**Tyrosol glucoside**	301,4b	159,4a	67,7a	72,9a	226,7b	199,8a
**Demethyl oleuropein**	0,0a	0,0a	0,0a	0,0a	191,6a	122,8a
**Oleuropein**	6817,7a	13917,2b	5607,3a	5305,6a	3280,9a	3223,0a
**Comselogoside**	2010,7a	2253,9b	3867,9b	1556,5a	646,1a	893,9b
**Ligstroside**	218,1a	834,0b	390,8b	312,4a	130,9a	136,9a
**Verbascoside**	1437,5a	3432,7b	1230,5a	2554,4b	3298,9a	3193,6a
**Rutin**	216,0a	381,3b	593,7b	149,1a	427,7a	390,9a
**luteolin-7-glucoside**	182,6a	264,8b	60,6a	155,9b	587,2a	547,4a
**total**	14304,1a	23114,3b	12600,4b	11198,5a	11962,8a	11484,8a

(*) mean contents and standard deviations from three different analyses

(**) different letters within the same olive cultivar indicate statistically significant differences ($p \leq 0.05$)

Table S6. Real Time-Quantitative PCR primers used in this work.

Primer	Sequence (5′ → 3′)
qOeGLU1A-F	GAAGAACGTCGTAAAAGGCT
qOeGLU1A-R	CAACAATGACATGACTTTTCAACC
qOeGLU1B-F	GAAGAACGTCGTAAAAGGCT
qOeGLU1B-R	GTTGAAACCTCACTGGCCAGGTCTTT
qOePPO1-F	AGTGTACTGCTGCCGGAAGTTTG
qOePPO1-R	TGTTGATTTGGAACCAACCACCT
qOePPO2-F	CGCCTGCTGTTCTTGTTTTCTCA
qOePPO2-R	GTGCAAAGTGCAACCATCGATTA
qOe1LOX2-F	GAGAATTGGGTGCGTTCATAC
qOe1LOX2-R	TCCTCTGGTGTGGCTAATGTC
qOe2LOX2-F	GATCCAAACGACACCGAAAAGGAGGAA
qOe2LOX2-R	GGCATTTATATACTTATCATCTTTCCAG
qOeEF1α-F	TGCTCTATCTGGATTGCCATT
qOeEF1α-R	TCAAATGCCACCATGACTTC
qOeGAPDH-F	TGAGATGCTGCACAATGGTT
qOeGAPDH-R	CACGATAGGCTTACGCAACA
